# Whole-brain functional activity and connectivity for the classification of subjective tinnitus: a machine learning study

**DOI:** 10.3389/fneur.2026.1796906

**Published:** 2026-08-07

**Authors:** Jianxiong Song, Fang Ouyang, Yongqiang Shu, Pengfei Yu, Xin Peng, Tong Wang

**Affiliations:** 1 Department of Otolaryngology, Jiangxi Provincial Children’s Hospital, Nanchang, China; 2 Department of Endocrinology, The First Affiliated Hospital of Nanchang University, Nanchang, China; 3 Department of Radiology, The First Affiliated Hospital of Nanchang University, Nanchang, China; 4 Department of Artificial Intelligence and Informatization, The Second Affiliated Hospital of Nanchang University, Nanchang, China

**Keywords:** logistic regression, machine learning, resting-state functional magnetic resonance imaging, subjective tinnitus, support vector machine

## Abstract

**Background and purpose:**

Clinical evaluation of subjective tinnitus mainly depends on patients’ self-reported auditory complaints, and standardized neuroimaging biomarkers for characterizing its central brain functional abnormalities remain lacking. The aim of this study is to utilize the resting-state functional magnetic resonance imaging (rs-fMRI) machine learning technique based on the region of interest (ROI), and to construct an exploratory classification framework for subjective tinnitus by analyzing functional activities and connectivity.

**Methods:**

The rs-fMRI data of 63 patients with subjective tinnitus (38.79 ± 15.79) and 84 healthy controls (HCs) (42.01 ± 9.45) were collected from the Department of Otorhinolaryngology, the first affiliated Hospital of Nanchang University. Five analysis methods were used: regional homogeneity (ReHo), amplitude of low frequency fluctuation (ALFF), fraction amplitude of low frequency fluctuation (fALFF), resting state functional connectivity (RSFC) and degree centrality (DC). A total of 7,134 features are extracted after *z* conversion. Then, the predicted features were selected through Mann–Whitney *U* test, the variables with high pairwise correlation (the correlation coefficient is greater than 0.75) were removed, the least absolute shrinkage and selection operator method was used to screen the features. Finally, a machine learning model was constructed by combining logistic regression (LR), support vector machine (SVM) and random forest (RF), and the performance differences of the three models were compared.

**Results:**

21 features are retained, including 3 zRSFCs, 1 zALFFs, 6 zfALFFs, 3 zDCs, and 8 zReHos. Based on these 21 features, the model accuracy and area under the curve constructed by LR, SVM and RF were 75.51% and 0.80, 80.27% and 0.82, 73.47% and 0.79, respectively.

**Conclusion:**

Our findings indicate that the ROI-based rs-fMRI machine-learning provides preliminary proof-of-concept evidence for the objective confirmation of subjective tinnitus. The imaging information based on rs-fMRI has the potential to become a neuroimaging biomarker for tinnitus.

## 1 Introduction

Tinnitus is the perception of sound in the absence of any external sound stimulus. Globally, 15% of people have experienced tinnitus (1), among whom approximately 20% of adults have significant symptoms, accompanied by depression, cognitive dysfunction, and sleep disorders, etc. (2). Tinnitus can be associated with a variety of factors, such as ear diseases, exposure to noise, hearing loss and aging (3). There is a high degree of heterogeneity among patients in terms of their experience of tinnitus and their response to treatment. Therefore, the uncertainty of the mechanism of tinnitus and the heterogeneity of symptoms affect the diagnosis and treatment of patients. Early identification and intervention are of great importance.

Both animal experiments and clinical studies have shown that there are central nervous system changes related to tinnitus (2, 4). These changes involve different locations of the auditory pathway, the auditory cortex, and non-auditory brain regions (5). On a sensory level, it is believed that tinnitus is caused by damage to the cochlea. However, many patients with tinnitus have normal hearing tests and there are no direct signs of cochlear damage (6). Even if the auditory nerve is severed, tinnitus cannot be eliminated, indicating that tinnitus may not be influenced by the activity of the auditory nerve (7). Some scholars also believe that tinnitus originates from the auditory brainstem (the cochlear nucleus). The pathological changes in the spontaneous neuronal activity within the auditory brainstem can drive the reorganization of the auditory cortex (8). On a macroscopic level, tinnitus is associated with abnormal oscillations in brain activity (9). Weisz et al. (10) discovered that in patients with tinnitus, the power of the *δ* and *γ* frequency bands in the temporal region increased, while the activity of the *α* band decreased. This is highly consistent with the model of thalamocortical rhythm disorder (11). The decrease in *α*-band power is related to cortical excitability (12). In patients with chronic tinnitus, asynchronous release of *α* was observed, which promoted neuronal activity. Of course, these activities are not limited to the auditory cortex; tinnitus is the result of the joint participation of various brain regions.

In clinical practice, the diagnosis of tinnitus is mainly based on the patient’s complaint and the collection of medical history. Imaging examinations are essentially an exclusionary test that identifies the objective causes that may cause tinnitus. For subjective tinnitus, there is currently no objective test available in clinical practice to observe changes in brain activity related to tinnitus. Accordingly, it is meaningful to develop objective imaging analytical approaches to characterize central functional abnormalities in individuals with subjective tinnitus. Compared to other neuroimaging tools, functional magnetic resonance imaging (fMRI) has great potential to change clinical practice because it can integrate anatomy, function and imaging, and has the advantages of being non-invasive, non-radiogenic and easily repeatable. The researchers conducted extensive studies on the functional changes in the brain regions related to tinnitus and achieved certain results. They discovered some possible imaging markers. However, these studies merely compared the inter-group differences between the tinnitus group and the control group to uncover information related to the occurrence of tinnitus, and they cannot be used for the prediction of tinnitus. If the fMRI data are combined with advanced analysis algorithms, it is expected to reveal the unique neural characteristics of subjective tinnitus.

After entering the new era of precision medicine, by extracting high-dimensional qualitative or quantitative features from clinical images and clinical data, and identifying the correlations among these information, it is possible to support evidence-based clinical decision-making (13). Machine learning is an artificial intelligence approach that is achieved through self-learning computer software. It is a promising method for extracting features from large information sources and has an advantage in handling datasets that contain complex nonlinear patterns (14). By manually extracting the features of brain signals and combining them with machine learning or deep learning models, discriminative information can be provided for neurological classification tasks (15). In recent years, many scholars have conducted research on artificial intelligence based on MRI in the field of neurological diseases. Golkarieh et al. (16) utilized a public 2D brain MRI dataset to conduct a comparative analysis of multiple deep learning and Transformer models for the diagnosis of multiple sclerosis, and demonstrated excellent performance. Raeisi et al. (17) have focused on artificial intelligence research on tinnitus. By using brain-derived biomarkers and machine learning, they have objectively classified tinnitus and have demonstrated a wealth of possibilities to us. Liu et al. (18) collected resting-state fMRI (rs-fMRI) data of 187 patients with attention deficit/hyperactivity disorder and 187 healthy controls (HCs). They combined these data with parameters such as regional homogeneity (ReHo) and low-frequency fluctuation amplitude (ALFF) along with a support vector machine (SVM) model, and found that the diagnostic accuracy for attention deficit/hyperactivity disorder reached 77.0%. Shi et al. (19) extracted radiomics features by averaging the amplitudes of low-frequency fluctuations in the images, and analyzed these feature information using machine learning methods. The research results are helpful for the early diagnosis of Parkinson’s disease and provide support for the study of the mechanism of Parkinson’s disease and its clinical assessment. However, few existing studies have integrated machine learning and neuroimaging to explore candidate neuroimaging biomarkers for identifying brain functional alterations associated with subjective tinnitus.

As far as we know, this is the first study to incorporate multiple imaging biomarkers into subjective tinnitus using rs-fMRI. We recruited 63 subjective tinnitus patients (STs) and 84 HCs. We extracted imaging biomarkers using five preprocessed rs-fMRI indicators, including ALFF, fractional ALFF (fALFF), degree centrality (DC), ReHo, and resting-state functional connectivity (RSFC), for quantitative analysis of tinnitus.

We constructed three machine learning models including logistic regression (LR), SVM, and random forest (RF) to differentiate patients with subjective tinnitus from healthy controls. Our core hypothesis was that rs-fMRI-derived brain functional features combined with machine learning could yield preliminary classification performance to separate STs from healthy participants within our single-center cohort.

## 2 Materials and methods

### 2.1 Participants

This is a prospective study that is intended to include appropriate tinnitus patients. Inclusion criteria: (1) the diagnosis of idiopathic tinnitus accords with the consensus of tinnitus experts; (2) over 18 years old; (3) right-handed; (4) external auditory canal is unobstructed, tympanic membrane intact; (5) the ability to express and understand is normal. Exclusion criteria: (1) MRI contraindications; (2) severe central system diseases or other systemic diseases such as cardiovascular diseases; (3) craniocerebral trauma, history of ear surgery, etc.; (4) Meniere’s disease, acoustic neuroma, etc.; (5) external otitis, otitis media, etc.; (6) history of drug addiction; (7) history of mental illness and medication use. From July 2022 to December 2023, a total of 63 STs were treated in the first affiliated Hospital of Nanchang University. Eighty-four HCs without tinnitus and with normal hearing were included, and they were matched with STs in terms of age, sex, education, and habitual hand. This study was approved by the Ethics Committee of the first affiliated Hospital of Nanchang University [(2022) medical research review no. (1–016)], and all the participants signed informed consent forms.

The clinical data of the participants were collected, including their age, sex, years of education, and disease duration. Under the guidance of the physician, we asked the patient to carefully and completely fill out the tinnitus handicap inventory (THI) and the tinnitus evaluation questionnaire (TEQ).

We conducted pure-tone audiometry, acoustic immittance tests and tinnitus matching tests on STs. Patients with abnormal hearing were not included in the study. At the same time, we used the tuning fork test and otoscope examination to ensure that the hearing of the HCs was generally normal.

### 2.2 Rs-fMRI data acquisition

All participants had their MRI images collected using a 3.0 Tesla MRI scanner equipped with an 8-channel phased-array head coil (Siemens, Erlangen, Germany) at our hospital. Foam pads and earplugs were employed to reduce patients’ head movements and minimize the scanner’s noise. Prior to the scan, participants were instructed to keep their eyes closed while refraining from falling asleep. Additionally, these participants were further directed not to engage in any specific cognitive activities during data acquisition.

Rs-fMRI data were acquired via a gradient-recalled echo planar imaging (EPI) sequence in the axial plane (repetition time = 2000 ms; echo time = 30 ms; flip angle = 90°; slice thickness = 4.0 mm; inter-slice gap = 1.2 mm; field of view = 230 mm × 230 mm; matrix size = 64 × 64; number of slices = 30), and a total of 240 rs-fMRI images were recorded.

The MRI data collection for this project was carried out at the Clinical Research Center for Medical Imaging in Jiangxi Province (No. 20223BCG74001).

### 2.3 Data preprocessing

The rs-fMRI data were examined using the MRIcro software and any data of poor quality were discarded. The data preprocessing was based on SPM12 and MATLAB2018b. Firstly, the file format was converted from DICOM to NIFTI, and the first 10 time points were deleted. Then, participants with the maximum base displacement (*x*, *y*, *z*) > 2.5 mm, the maximum spin (*x*, *y*, *z*) > 2.5°, or any volume with a frame-wise displacement (FD) exceeding 2.5 standard deviations were excluded. The functional images of all participants were co-aligned with the mean of the realigned EPI images, and segmented into gray matter, white matter, and cerebrospinal fluid. After head motion correction, the functional images were registered to the Montreal Neurological Institute (MNI) space using the DARTEL tool, and then resampled to a resolution of 3 mm × 3 mm × 3 mm; the standardized images were smoothed with a Gaussian kernel of 6 mm × 6 mm × 6 mm. Finally, the Friston 24 parameters, white matter signal, and cerebrospinal fluid signal were regressed out from the time series of all voxels using a linear regression method. The selected frequency band was 0.01–0.08 Hz to improve the signal-to-noise ratio.

### 2.4 Feature extraction

The rs-fMRI features were calculated using the PyRadiomics package based on Python 3.7 in PyCharm.^1^ From each of the five original images of ReHo, ALFF, fALFF, DC and RSFC, we extracted features in 116 brain regions (90 major brain regions and 26 minor brain regions) delineated by the anatomical automatic labeling (AAL) atlas.

ReHo: We further applied band-pass filtering, with the frequency band selected as 0.01–0.08 Hz; we then performed the ReHo calculation and smoothed it using a Gaussian kernel of 6 mm × 6 mm × 6 mm, thereby obtaining the ReHo values for the entire brain.

ALFF/fALFF: After smoothing with a Gaussian kernel of 6 mm × 6 mm × 6 mm, the ALFF/fALFF calculation was carried out, with the frequency band selected as 0.01–0.08 Hz.

DC: Firstly, we performed band-pass filtering with the frequency band selected as 0.01–0.08 Hz; we then performed the DC calculation, with the correlation threshold set at 0.25, and smoothed it using a Gaussian kernel of 6 mm × 6 mm × 6 mm.

RSFC: First, a band-pass filter (0.01–0.08 Hz) and smoothing (6 mm × 6 mm × 6 mm Gaussian kernel) were performed. Based on the AAL template, we divided the 116 brain regions into regions of interest and calculated the pairwise Pearson correlations between them. Finally, 6,670 zRSFC values were selected.

Ultimately, we obtained a total of 7,134 measurement values, including 116 zsReho, 116 zALFF, 116 zfALFF, 116 zDC, and 6,670 zRSFC values. These measurement values will all be included as categorical variables in the subsequent model construction.

### 2.5 Construction of machine learning model

For the small sample cohort, in order to maximize the utilization rate of the samples, this paper employed the Leave-One-Out (LOO) cross-validation method for model training and evaluation. Firstly, we identified the features that showed differences between the two groups through the Mann–Whitney test (*p* < 0.1). Secondly, we calculated the Pearson correlation coefficients between the features and set the absolute value threshold of the correlation coefficient at 0.75. For feature pairs with a correlation greater than the threshold, we compared their respective average absolute correlations and deleted the feature with the larger average absolute correlation coefficient, thereby reducing multicollinearity at a lower cost. Then, we used the LASSO method for further feature selection. Finally, before model fitting, all features have been standardized using *z*-scores. Specifically, the StandardScaler from scikit-learn was fitted on the training set of each LOO fold. The resulting transformation was applied to both the training and test samples, and only the features with non-zero coefficients were selected for training the classification model. The feature selection was performed using the “scikit-learn” package in Python 3.8.8.

To prevent data leakage, all feature selection steps are nested within each LOO iteration. Specifically, in each fold, the test samples are completely excluded before feature selection or model training is carried out. The Mann–Whitney *U* test, correlation-based filtering, and LASSO feature selection are specifically applied to the training subset (146 samples). The resulting feature set and the trained model are then used to generate a single prediction that retains the test samples. This process is repeated for all 147 samples.

We used LR, SVM and RF to build classification models, and employed Kappa to evaluate the heterogeneity of the test results. The following are the corresponding parameters for three types of machine learning:

LR: penalty = ‘l2’, C = 0.1, max_iter = 1,000

SVM: kernel = ‘rbf’, C = 1.0, gamma = ‘scale’

RF: n_estimators = 100, max_depth = 10, min_samples_split = 2, min_samples_leaf = 1.

## 3 Results

### 3.1 General clinical data

The comparison of clinical data between STs and HCs is shown in Table 1. There is no significant difference between the two groups in age, sex, education level, handedness (*p* > 0.05).

**Table 1:** The clinical data of the participants.

Clinical indicators	STs (*n* = 63)	HCs (*n* = 84)	*p* value
Age (years)	38.79 ± 15.79	42.01 ± 9.45	0.052
Gender (male/female)	32/31	36/48	0.340
Education (years)	12.17 ± 3.05	11.24 ± 2.72	0.059
Handedness (right)	63	84	>0.99
Duration (years)	4.04 ± 4.01	–	–
TEQ	9.83 ± 3.32		
THI	43.21 ± 21.85		
Side (left/right/bilateral)	29/29/5	–	–

STs, subjective tinnitus patients; HCs, healthy controls; TEQ, tinnitus evaluation questionnaire; THI, tinnitus handicap inventory.

### 3.2 Feature extraction

**Figure 1:**
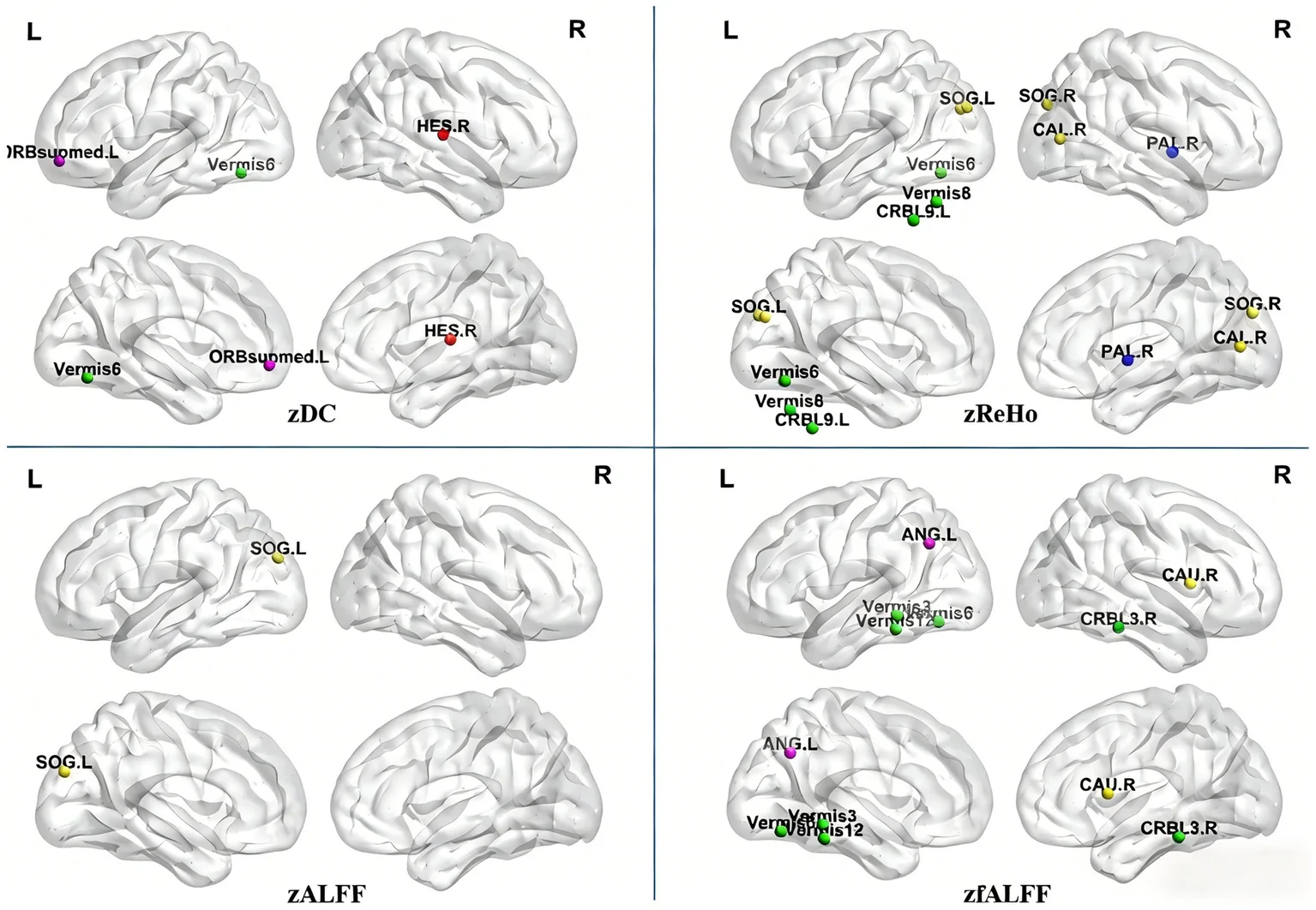
Visualized plots for selected zDC, zReHo, zALFF and zfALFF features. The color of the small balls represents the network to which the corresponding brain regions belong. Blue: auditory network; yellow: visual network; pink: default network; green: cerebellar network; red: other brain regions.

**Figure 2:**
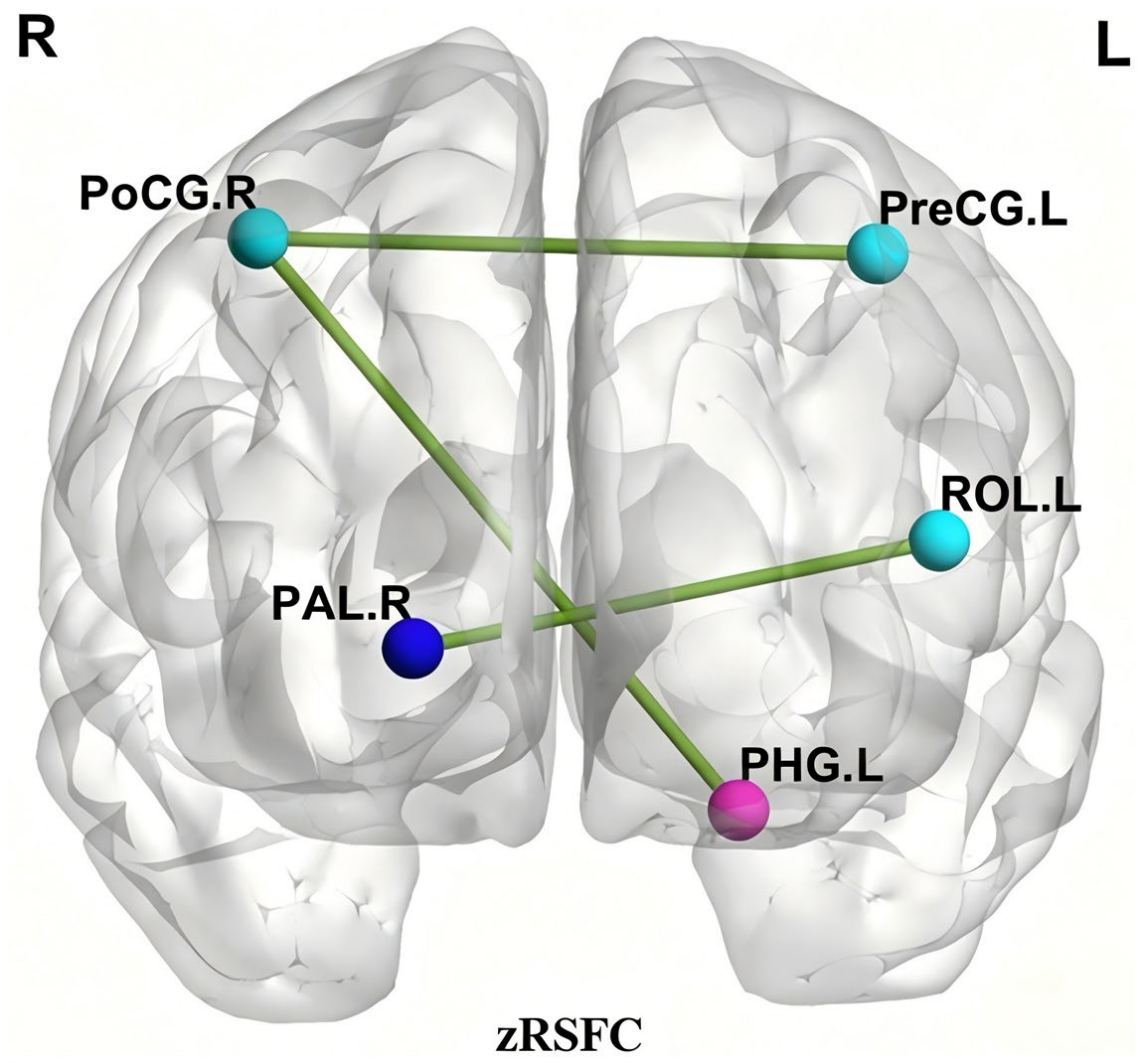
Visual plots of the selected zRSFC features. The color of the small balls represents the network to which the corresponding brain regions belong. Blue: auditory network; yellow: visual network; light blue: sensorimotor network; pink: default network; green: cerebellar network.

We conducted a stepwise Mann–Whitney *U* test on 7,134 features, removed variables with strong pairwise correlations (correlation coefficient greater than 0.75), and used the Least Absolute Shrinkage and Selection Operator (LASSO) to screen a total of 21 features (3 zRSFC, 1 zALFF, 3 zDC, 8 zReHo, and 6 zfALFF). Table 2 provides a detailed list of each feature with the AAL template, and these features are mainly located in the auditory network (AN), visual network (VN), default network (DMN), sensorimotor network (SMN), and cerebellar network (Table 2; Figures 1, 2).

**Table 2:** The rs-fMRI features used to distinguish STs from HCs.

ID	Feature	AAL number	Brain region	Network
1	RSFC	1–58	Precentral_L-Postcentral_R	SMN–SMN
2	RSFC	17–76	Rolandic_Oper_L-Pallidum_R	SMN–AN
3	RSFC	58–39	Postcentral_R-ParaHippocampal_L	SMN–DMN
4	DC	25	Frontal_Mid_Orb_L	DMN
5	DC	80	Heschl_R	Other region
6	DC	112	Vermis6	Cerebellum
7	Reho	44	Calcarine_R	VN
8	Reho	45	Cuneus_L	VN
9	Reho	49	Occipital_Sup_L	VN
10	Reho	50	Occipital_Sup_R	VN
11	Reho	76	Pallidum_R	AN
12	Reho	105	CRBL9.L	Cerebellum
13	Reho	112	Vermis6	Cerebellum
14	Reho	114	Vermis8	Cerebellum
15	ALFF	49	Occipital_Sup_L	VN
16	fALFF	65	Angular_L	DMN
17	fALFF	72	Caudate_R	VN
18	fALFF	96	CRBL3.R	Cerebellum
19	fALFF	109	Vermis12	Cerebellum
20	fALFF	110	Vermis3	Cerebellum
21	fALFF	112	Vermis6	Cerebellum

AAL, automatic anatomical labeling; ALFF, amplitude of low frequency fluctuation; fALFF, fraction amplitude of low frequency fluctuation; DC, degree centrality; ReHo, regional homogeneity; RSFC, resting-state functional connectivity; AN, auditory network; SMN, sensorimotor network; DMN, default network; VN, visual network.

### 3.3 Machine learning analysis

**Figure 3:**
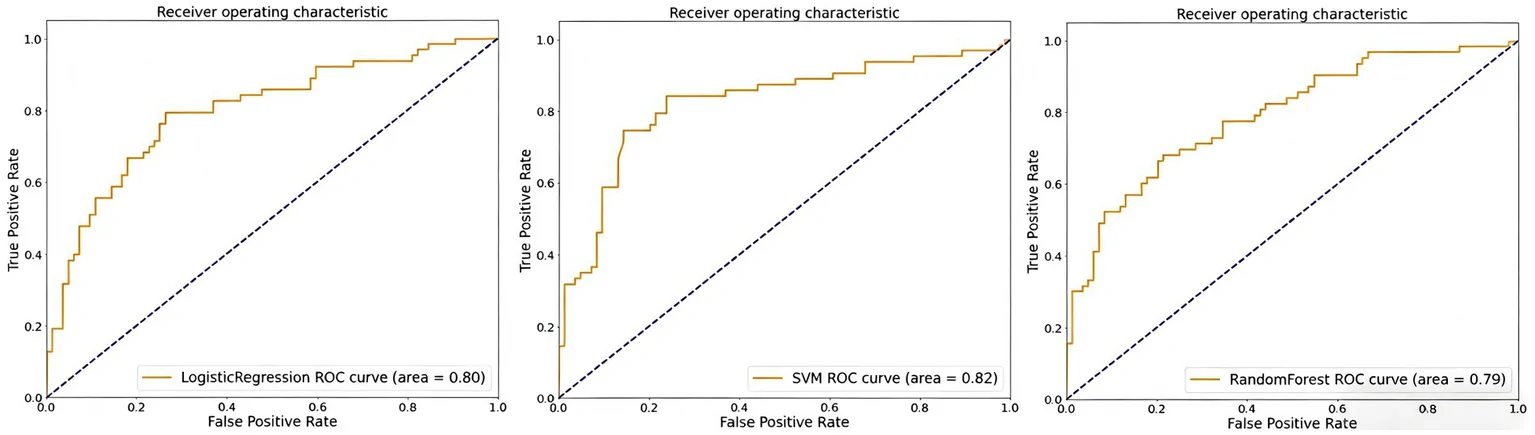
Efficacy of three machine learning methods for classification model construction.

After training and validation using the LOO method, we comprehensively compared the model indicators to select the best model. The three models were ranked by the area under the curve (AUC) as follows: LR (0.80), SVM (0.82), and RF (0.79) (Table 3; Figure 3). The results show that the 21 selected features are feasible for distinguishing STs from HCs, and among them, SVM has the best performance in classification.

**Table 3:** The performance of four types of machine learning.

Machine learning	Accuracy	Sensitivity	Specificity	AUC	Kappa
LR	75.51%	77.78%	73.81%	0.80	0.51
SVM	80.27%	73.02%	85.71%	0.82	0.60
RF	73.47%	66.67%	78.57%	0.79	0.46

LR, logistic regression; SVM, support vector machine; RF, random forest.

## 4 Discussion

This exploratory study constructed an region of interest (ROI)-based rs-fMRI machine learning classification framework to extract resting brain functional features and distinguish STs from HCs. Our internal cross-validation results demonstrated that the model could capture abnormal rs-fMRI signals linked to subjective tinnitus, providing preliminary neuroimaging evidence to support central neural dysfunction in tinnitus patients. Further multi-center external validation is required to confirm its translational potential before clinical application.

The 21 rs-fMRI features obtained from the analysis were input into LR, SVM and RF to construct classification models. The AUC rankings obtained are as follows: SVM (0.82), LR (0.80), RF (0.79). Overall, the performance of these three machine learning methods is broadly comparable, with SVM showing numerically higher performance.

For some dynamic and complex diseases in clinical practice, using nonlinear physiological features and machine learning classification for prediction is of great significance. Shamsi et al. (20) extracted complex nonlinear features and conducted classification through probabilistic neural networks for electrocardiogram signals, achieving highly accurate prediction of the outcome of external defibrillation for patients with out-of-hospital cardiac arrest. Moreover, prediction modeling can also be applied in the field of medical diagnosis, providing precise diagnoses for individual patients and reducing unnecessary costs. In recent years, many efforts have been made to combine neuroimaging and machine learning to implement complex classification procedures. The accuracy rate of machine learning classification performance varies widely, ranging from 71.0 to 99.4% (21–23). Among them, the highest performance was achieved by quantum neural networks (with an accuracy rate of 99%) (24). Liu et al. (25) included 46 STs and 56 HCs. For each subject, 61 features were extracted to train the SVM model. The accuracy rates of the training dataset and the test dataset were 80.49 and 80.00% respectively, and the AUC was 0.86. However, when combining the wavelet scattering transformation features with clinical characteristics, the AUC reached 92.53%, the sensitivity was 84.84%, and the specificity was 83.04% (26). In another study on the treatment of tinnitus, Cardon et al. (23) used an RF classifier to predict the prognosis of tinnitus symptoms alleviated by transcranial direct current stimulation, with an accuracy rate of 58.71%. Since the input data is readily available, this model has the potential to be easily implemented and evaluated in clinical settings. These research findings indicate that more relevant information can be obtained from independent brain regions, and by combining machine learning methods, a new perspective has been opened up for the objective confirmation and prognosis of tinnitus.

When auditory input decreases, such as after damage to the cochlea, the neural auditory response may alter the cortical circuits and functions. Chronic subjective tinnitus is an example of this. The dysfunction of the neuronal plasticity in this condition causes only the sounds that the patient perceives to be generated (27, 28). These maladaptive responses may include the release of sensory input and lateral inhibition, as well as irregular spontaneous functional activities within the neural network related to sound processing (29). Many important structures in the auditory network, such as the cochlear nucleus and the inferior colliculus, may be related to the initial generation of tinnitus signals. A recent fMRI study revealed that the ReHo of the primary auditory cortex decreased (30), while the ReHo of some secondary auditory regions increased. Additionally, there is much evidence suggesting that tinnitus is related to functional disorders of the entire auditory system (31, 32). The AN not only serves as a key area for differentiating STs from HCs, but more importantly, it may be the initiating factor for tinnitus. Furthermore, abnormal patterns of brain activity in patients with tinnitus have been identified in the auditory pathways including the auditory cortex, as well as in non-auditory regions such as the prefrontal cortex (33). In combination with our research findings, tinnitus perception may be generated by the activities of networks overlapping and separate in the auditory system and multiple parts, including the VN, DMN, SMN and cerebellar networks.

The VN is mainly composed of the occipital cortex on both sides of the calcarine sulcus, responsible for spatial position perception, transmission and processing of visual information. Our results show that the calcarine sulcus is a characteristic that can distinguish the two. As an important component of the primary visual cortex, it is the main area for receiving signal inputs from the retina. There have been many studies on the changes in VN in previous tinnitus research. Chen et al. (34) found that the ALFF values in the visual area of patients with chronic tinnitus were decreased. Maudoux et al. (35) discovered functional connections between the auditory and visual networks in tinnitus patients. This might be due to the compensatory mechanism related to the VN associated with hearing the tinnitus hallucinations. The multisensory connection between the auditory and visual areas enables the perception of tinnitus to alter the brain activity in the visual area. What specific role VN plays in the generation of subjective tinnitus remains to be explored, but it is beyond doubt that VN has great potential and value in identifying STs and HCs.

The DMN is a brain system whose activity increases during the resting state and decreases during the task state (36). The DMN is generally believed to consist of the posterior cingulate cortex, the precuneus, the anterior cingulate cortex, the angular gyrus, and the medial prefrontal cortex (37), and plays an important role in emotion control and processing of painful information. Since most patients with tinnitus have negative emotions such as anxiety and depression, some scholars have proposed that the occurrence of tinnitus may be related to the abnormality of the DMN (38). Our results show that there are local indicators (DC/fALFF) and functional connectivity abnormalities within the DMN. Similarly, functional abnormalities in the DMN of STs have been widely confirmed. A study using graph theory analysis demonstrated that the functional connectivity between the DMN and other brain networks in tinnitus patients was enhanced (39). Moreover, it was found that the connectivity between the DMN and the limbic system was related to tinnitus distress (40). The precuneus, in particular, can serve as a marker for long-term tinnitus and its severity (41). Neurofeedback training within the DMN can alleviate the pain associated with tinnitus (42), further illustrating the role of the DMN in tinnitus. These findings help us better understand the mechanism of tinnitus distress and contribute to the treatment and intervention of tinnitus.

In previous studies, SMN was rarely mentioned, but the brain regions it encompasses (such as the central prefrontal cortex, central posterior cortex, etc.) are presumably quite familiar. The abnormal characteristics of SMN in the results mainly focused on RSFC, which is similar to many previous studies on tinnitus (43–45). The SMN plays a crucial role in the brain, primarily processing sensory stimuli such as touch, vision, and hearing and initiating corresponding physical responses, as well as coordinating the cerebellum and basal ganglia to plan and execute movements. Head and neck movements or muscle contractions can either trigger or suppress tinnitus, or affect the loudness of tinnitus (46). This “somatic sensory tinnitus” is considered a common subtype of tinnitus, suggesting that abnormal movements of the head and neck muscles may be associated with abnormal SMN. Panouillères and Möttönen (47) proposed the auditory-motor decline hypothesis, suggesting that the defect of the auditory system leads to a reduction in the recruitment of the motor system during speech processing in patients with hearing loss. In a study on the cognitive dysfunction associated with tinnitus, it was found that the poor results of the Montreal Cognitive Assessment in patients with tinnitus accompanied by hearing loss and cognitive dysfunction were related to the interruption of the SMN (48). We speculate that tinnitus is a proprioceptive illusion, associated with widespread emotional and somatic sensory dysfunction. The observed abnormality of SMN may mainly be caused by hearing loss (49). These studies not only provide us with a deeper understanding of the role of SMN in the tinnitus network, but also may be helpful in differentiating STs from HCs.

It is worth noting that a significant portion of the imaging biomarkers selected in our study originated from the cerebellar network. Increasing evidence suggests that the cerebellum is not only crucial for motor learning and coordination, but also plays an important role in cognition and emotion. Some cerebellar regions can receive input from the auditory center (50) and are activated by both simple and complex sounds (51). There may be several potential mechanisms by which the cerebellum network is involved in tinnitus. Animal studies on tinnitus have shown that the flocculonodular lobe of the cerebellum, as a gain control mechanism, compares the input from the cochlea with the descending signals from the cerebral cortex (52), regulating the sensory or emotional characteristics of tinnitus. Petacchi et al. (53) found that the cerebellum is a key area for collecting sensory data and pitch recognition, so the “noise” related to tinnitus may be due to the failure to distinguish and encode the information of different pitch sounds, or due to some mismatch in neural activities. The central gain mechanism of the cerebellum can highly specifically filter out repetitive and unwanted noises, preventing these noises from reaching the auditory cortex (8). Once the cerebellum-brain functional connection is disrupted, tinnitus may be perceived and undergo long-term reorganization in the auditory cortex, turning into a chronic condition. In the future, when dealing with subjective tinnitus, we should pay attention to the cerebellar network.

## 5 Limitations

This study has several significant limitations that need to be acknowledged. Firstly, all participants with subjective tinnitus underwent a complete audiological examination, while the healthy control group only received qualitative hearing screening through otoscope and tuning fork tests, without quantitative pure-tone audiometry. This means that mild subclinical hearing loss cannot be completely ruled out as a potential confounding factor affecting brain functional imaging signals. Secondly, this dataset only includes STs and HCs, and does not include objective tinnitus or other clinical populations with tinnitus-like symptoms. Therefore, the machine learning model we constructed lacks the ability to differentiate and diagnose heterogeneous hearing impairments. Thirdly, this is a single-center exploratory analysis that relies solely on internal cross-validation; an external independent cohort is needed to verify the generalizability of the extracted imaging features and classification models. Moreover, the focus of this study is to explore the central nervous signals related to subjective tinnitus, rather than developing mature tools for routine clinical diagnosis. In the future, with sufficient funds, multi-center research will increase the sample size, conduct comprehensive audiological evaluations for all participants, cover multiple clinical subgroups, build various classification models, and carry out external validation to further overcome these limitations and enhance its translational application value.

## 6 Conclusion

This preliminary proof-of-concept study demonstrates that an ROI-based rs-fMRI machine learning pipeline achieves promising classification performance for differentiating STs from HCs within our single-center cohort. Nevertheless, the lack of independent validation cohorts limits the generalizability of our findings, and further multi-cohort verification is mandatory before exploring its clinical translational value. Moreover, ROI-based resting-state imaging metrics capture brain functional changes linked to the central neuropathology of subjective tinnitus, offering valuable imaging features to support subsequent research unpacking the neural mechanisms of tinnitus.

## Data Availability

The raw data supporting the conclusions of this article will be made available by the authors, without undue reservation.

## Footnotes

1
https://www.jetbrains.com/pycharm/
