# Supplementary material for: Whole-brain functional activity and connectivity for the classification of subjective tinnitus: a machine learning study
Source: Front Neurol. 2026 Aug 7;17:1796906. doi: 10.3389/fneur.2026.1796906 (PMC13493256; doi:10.3389/fneur.2026.1796906)

Supplementary Table 1 | The rs-fMRI features used to distinguish STs from HCs

ID	Feature	AAL Number	Brain region	Network	Feature selection frequency
1	RSFC	1-58	Precentral_L-Postcentral_R	SMN-SMN	147/147
2	RSFC	17-76	Rolandic_Oper_L-Pallidum_R	SMN-AN	147/147
3	RSFC	58-39	Postcentral_R-ParaHippocampal_L	SMN-DMN	147/147
4	DC	25	Frontal_Mid_Orb_L	DMN	147/147
5	DC	80	Heschl_R	Other region	147/147
6	DC	112	Vermis6	celebellum	147/147
7	Reho	44	Calcarine_R	VN	147/147
8	Reho	45	Cuneus_L	VN	147/147
9	Reho	49	Occipital_Sup_L	VN	147/147
10	Reho	50	Occipital_Sup_R	VN	147/147
11	Reho	76	Pallidum_R	AN	147/147
12	Reho	105	CRBL9.L	celebellum	147/147
13	Reho	112	Vermis6	celebellum	147/147
14	Reho	114	Vermis8	celebellum	147/147
15	ALFF	49	Occipital_Sup_L	VN	147/147
16	fALFF	65	Angular_L	DMN	147/147
17	fALFF	72	Caudate_R	VN	147/147
18	fALFF	96	CRBL3.R	celebellum	147/147
19	fALFF	109	Vermis12	celebellum	147/147
20	fALFF	110	Vermis3	celebellum	147/147
21	fALFF	112	Vermis6	celebellum	147/147

AAL, automatic anatomical labeling; ALFF, amplitude of low frequency fluctuation; fALFF, fraction amplitude of low frequency fluctuation; DC, degree centrality; ReHo, regional homogeneity; RSFC, resting-state functional connectivity; AN, auditory network; SMN, sensorimotor network; DMN, default network; VN, visual network

Supplementary Table 2 | Parameter combinations in machine learning

LR	SVM	RF
penalty='l2', C=1.0,max_iter = 1000	kernel = 'rbf', C =0.1, gamma = 'scale'	n_estimators = 100, max_depth = 10, min_samples_split = 2, min_samples_leaf = 1
penalty='l2', C=0.01,max_iter = 1000	kernel = 'rbf', C = 1.0, gamma = 'scale'	n_estimators = 200, max_depth = 20, min_samples_split = 2, min_samples_leaf = 1
penalty='l2', C=0.1,max_iter = 1000	kernel = 'rbf', C = 10, gamma = 'scale'	n_estimators = 200, max_depth = 5, min_samples_split = 10, min_samples_leaf = 5
	kernel = line, C = 1.0, gamma = 'scale'	

LR, logistic regression; SVM, support vector machine; RF, random forest

Supplementary Table 2

Machine-learning pipeline

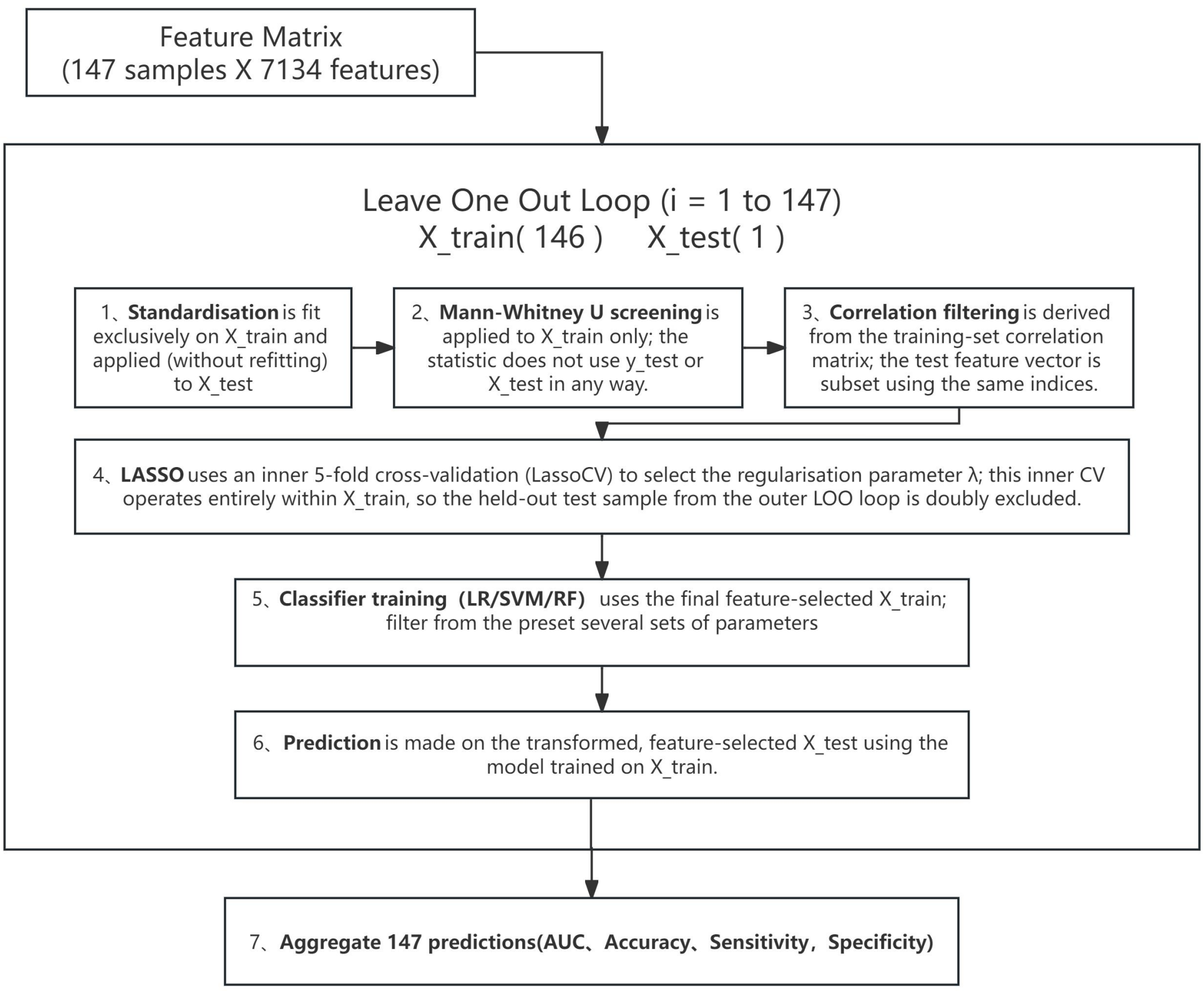

Supplement: Supplementary file 1 [file Data_Sheet_1.pdf]
